# Artificial intelligence in epidemic watch: revolutionizing infectious diseases surveillance

**DOI:** 10.3389/fdgth.2025.1692617

**Published:** 2025-12-04

**Authors:** Abdallah Borham, Lereen T. Kamal, Sungsoo Chun

**Affiliations:** Institute of Global Health and Human Ecology, School of Sciences and Engineering, The American University in Cairo, New Cairo, Egypt

**Keywords:** artificial intelligence, machine learning, infectious disease surveillance, public health policy, data analytics, pandemics

## Abstract

Artificial intelligence is undoubtedly emerging, and its various manifestations in technology are widely and deeply embedded in our communities. That is what obliges its mindful and skillful use and utilization, especially for infectious disease prevention. Over 1 million people are affected by infectious diseases, and the whole globe is carrying a huge burden of DALYs due to infection mortality and morbidity, which can be mitigated by the proper use of machine learning and deep learning features for data analytics and monitoring of real-time changes, and even point out the anticipated timing of pandemics. The application of machine learning and deep learning allows for forecasting and monitoring of outbreaks, which can contribute to converting the distribution of medical resources into an efficient, patient-centered approach. There are various algorithms and ML models applied in infectious disease surveillance to promote public health security. The following review considers the interface between AI and public health, with considerations of successful applications and concerns in technology acceptance and governance. Key public health policy recommendations derived from recent literature are also presented.

## 1 Introduction

The healthcare digitalization has resulted in an era where enormous amounts of information, now commonly referred to as big data, are being generated through electronic health records, surveillance systems for diseases, and monitoring devices for patients. In the US alone, the health care data are now estimated to have surpassed the zettabyte scale (10²¹ gigabytes) (1, 2). This data holds the huge potential for improving clinical decision-making, disease monitoring, and public health research, particularly when combined with the potential of Machine Learning (ML) and Artificial Intelligence (AI).

Infectious disease remains a persistent global health threat, and the burden of HIV, tuberculosis, and malaria disproportionately affects low- and middle-income countries (LMICs). Infectious diseases exhibit the familiar trajectory: animal-to-human transmission, to local epidemic, to eventual global spread, as occurred with the 1918 influenza pandemic and more recently in SARS-CoV-2 (3). Contributing factors such as climate change, urbanization, migration, and population aging have all contributed to the growth and transmission of infectious diseases. Urbanization, for example, has facilitated the spread of arboviral infections like Zika, dengue, and chikungunya, most of which are spread by Aedes aegypti and Aedes albopictus mosquitoes, which have been able to adapt to urban environments (4).

Traditional public health surveillance systems have long relied on statistical techniques to monitor and respond to outbreaks of disease (5, 6). AI and ML tools, nevertheless, now offer impressive enhancements. These tools possess the capacity to analyze complex datasets, detect epidemiological trends, and issue early alerts of outbreaks: attributes that are essential for effective and timely public health intervention (7, 8). For example, Lee et al. used six ML models, including support vector machines, random forests, and CatBoost, and 56 CDC parasite infection reports and PubMed case abstracts to predict malaria with 90% accuracy. The study identified nationality and travel destination as important pointers towards the diagnosis of the disease (9).

For all this enthusiasm, there are essential challenges. Implementing AI in public health requires robust data sources, advanced analytic models, awareness of ethical, social, and context-dependent subtleties. Decision-makers must also balance the credibility of disease modelling, social contact network analysis, and control measure evaluation: all of which require domain expertise and subtle comprehension (10). This review provides an overview of current uses of AI and ML for the detection, prediction, and surveillance of infectious diseases globally. It outlines successful case studies, reviews obstacles to implementation, and makes recommendations on policy options for maximizing the incorporation of AI within public health surveillance systems.

## 2 Inclusion and exclusion criterea

This policy and practice review discusses policy options and related areas of public health informatics research to overcome the challenges in hand while recommending reliable resolutions to them. By using a collaborative process, sections were assigned to the authors based on their areas of expertise. Each author conducted an independent review of the assigned literature, relying on concepts and keywords related to the research topic, and the final manuscript was evaluated to ensure consistency, coherence and coverage of the topic. Discrepancies in interpretation were resolved through discussion and consensus among all authors before finalizing the review.

A search strategy was applied to identify the relevant literature that tackled the applications of artificial intelligence (AI), machine learning (ML), and deep learning (DL) in infectious disease surveillance, prediction, and response. Predefined eligibility criteria were used.

The inclusion criteria were the following:

1. focus on AI/ML/DL for infectious disease surveillance, outbreak prediction, or pandemic preparedness.
2. publication in Peer-Reviewed Journals or authoritative public health reports
3. studies published in the last 15 years; and
4. articles in the English language only.

The exclusion criteria were the following:

1. irrelevant articles;
2. non-peer-reviewed Sources; and
3. articles in languages other than English.

One hundred articles were initially retrieved to cover this policy-related topic from both scholarly and non-scholarly perspectives, such as case studies, technical reports, public health policy briefs, and systematic reviews. After removing duplicates and conducting title and abstract screening, 52 articles were excluded due to irrelevance, methodological limitations, or lack of required data. The 48 articles included were thoroughly reviewed and included in the final policy review.

## 3 Global burden of infectious diseases

The global burden of disease (GBD) is the most comprehensive scientific effort to quantify the impact of diseases, injuries, and risk factors on populations (10). Infectious diseases—caused by bacteria, viruses, fungi, protozoa, and helminths (11, 12)—remain a considerable global health threat due to their potential to disseminate rapidly and result in high fatality. In 2019 alone, infectious diseases caused a total of approximately 1.3 million cases in China, accounting for 12.1% of the nation's disease burden (13). Globally, infectious diseases remain one of the leading causes of death, particularly in low-income countries and vulnerable populations such as children. In fact, two infectious diseases ranked among the top ten causes of death worldwide in 2019 (14). Infectious diseases can be broadly classified into three groups: those of high mortality, those causing long-term disability, and those with immense global spread potential. Mortality and morbidity measures are the quantitative rankings of disease burden, which cumulatively present the burden of disease (15, 16). The disease burden is usually expressed in Disability-Adjusted Life Years (DALYs)—a quantification of one year of healthy life lost due to premature death or disability (17).

**Figure 1:**
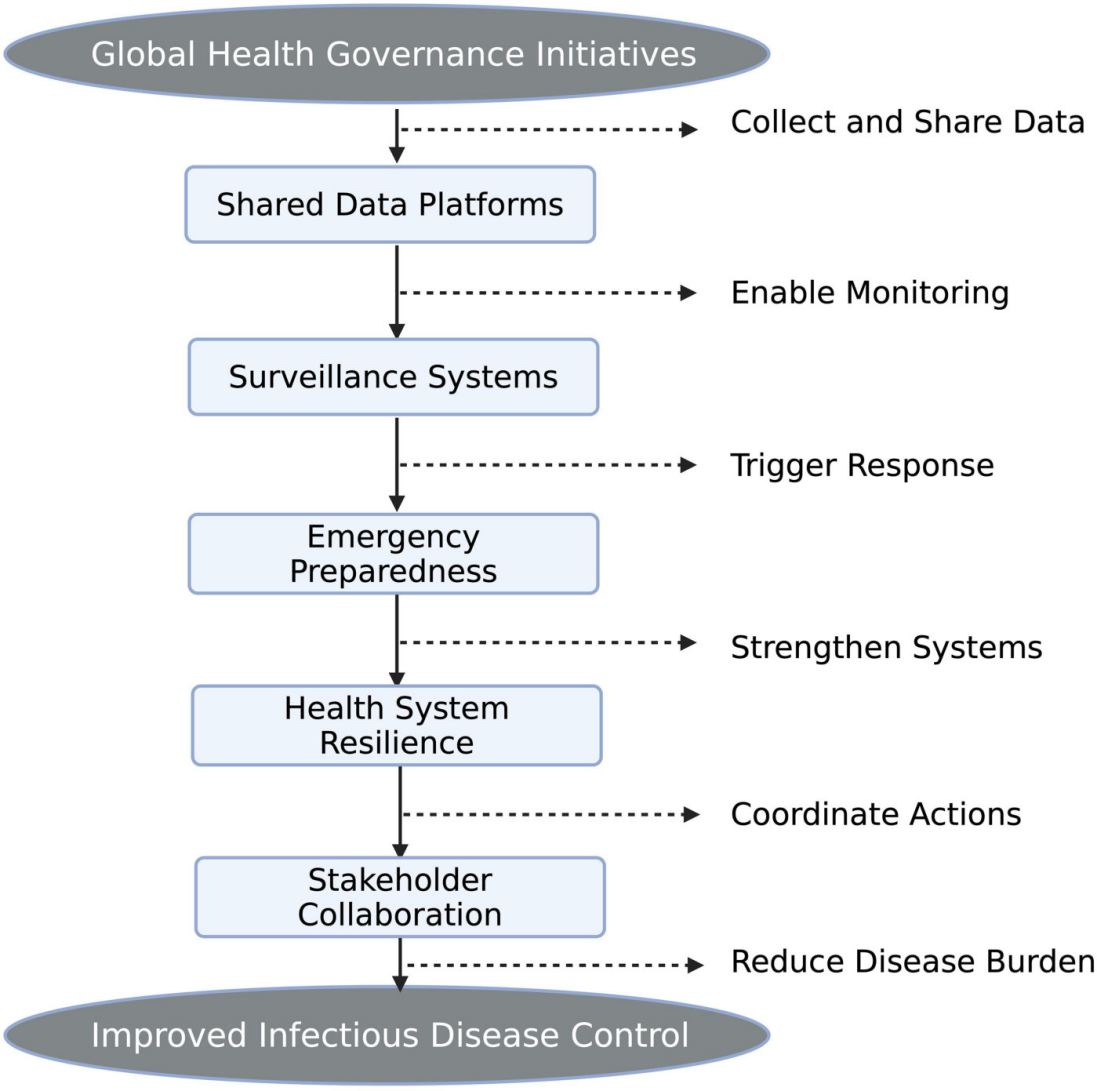
Global health governance in epidemic preparedness and systems resilience.

Addressing the infectious disease burden must be through strengthened global health governance. Initiatives now focus on building shared data platforms and tools for strengthening health system resilience. Some of the key features include emergency preparedness, such as rapid response and risk communication, and communicable disease surveillance to track trends in pathogens (13). System evaluation frameworks have also emerged as key for the production of data to assess and strengthen global disease control initiatives. Nevertheless, there are challenges to the governance of global health due to the growing number and influence of stakeholders in the field. And still, we didn't unlock the full potential of AI and how it could substantially assist in alleviating the global burden of disease, especially the infectious ones, as shown in Figure 1. In this review, we may not have fully accumulated that information; however, as it is a huge field and there's ongoing research, we believe in formulating such a notion (13).

## 4 AI/ML in surveillance and monitoring of infectious diseases

Artificial Intelligence (AI) refers to computer algorithms that simulate human intelligence, of which Machine Learning (ML) is a subset. ML entails various algorithms such as deep learning (DL), natural language processing (NLP), support vector machines (SVM), and artificial neural networks (ANN) through which systems can recognize patterns, make predictions, and draw conclusions from data (18).

**Figure 2:**
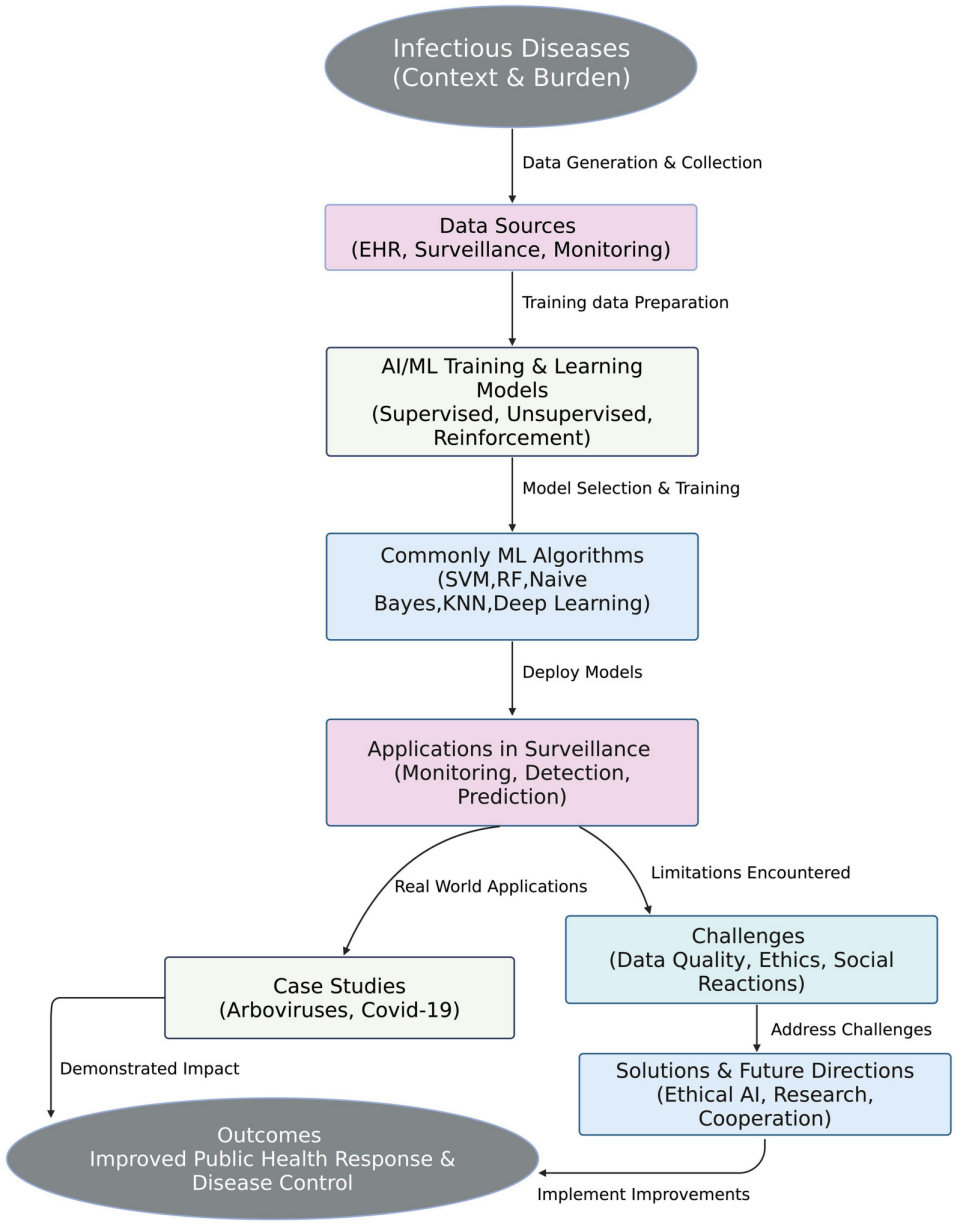
The impact of AI/ML technologies in improving outcomes and preventing outbreaks.

In disease surveillance, ML algorithms have been utilized to enhance surveillance by way of early detection of disease outbreaks, analysis of transmission dynamics, and predictive model building. DL can, for instance, be utilized for scanning radiological images for early indications of disease, and NLP can be utilized for the extraction of relevant information from unstructured scientific literature and clinical records, as shown in Figure 2 (19, 20).

### 4.1 Types of ML techniques in disease surveillance

#### 4.1.1 Supervised learning

It is achieved by training on labelled data with a known output. The approach is widely used for diagnosis classification, for example, cancer detection from images, and prognosis, for example, disease recurrence prediction from Electronic Health Records (21, 22). Supervised models have also forecasted outcomes like malaria incidence and outbreak timing in infectious disease contexts accurately using structured data.

#### 4.1.2 Unsupervised learning

It analyzes data without labelled data, and therefore, it applies to risk clustering, anomaly detection, and pattern discovery. In healthcare, it can identify latent disease subtypes or forecast disease susceptibility based on genomic data. Unsupervised learning saves money when outcome labels are not available or expensive to obtain (21, 22). Semi-supervised learning is a combination of the two and is most relevant in cases with minimal labelled data.

#### 4.1.3 Reinforcement learning

It allows models to learn through experience, interacting with the environment, and receiving feedback. Although more common in automation and robotics, its applications within public health are streamlining treatment pathways and augmenting intervention planning in adaptive health systems (21, 22).

#### 4.1.4 Deep learning

It utilizes multi-layered neural networks in identifying complex patterns in massive sets of data. In public health, DL has driven disease modelling, disease forecasting, and even the detection of mosquito vectors for malaria surveillance (21, 23). Neural network hidden layers enable the segmentation of complex epidemiological data into segments that can be studied (24).

#### 4.1.5 Natural language processing (NLP)

It allows rapid processing of big text data. In infectious disease monitoring, NLP has been used to review clinical notes, discover trends from literature studies, and monitor emerging dangers by monitoring the media reporting (21, 23).

### 4.2 Machine learning applications in infectious disease surveillance

Machine learning has proven to have robust applications in malaria monitoring. ML models have used data from various sources like climate markers, densities of mosquitoes, and case reports to predict malaria epidemics with high accuracy (1, 25, 26). For example, Bayesian networks and ANN models have been able to effectively combine satellite-derived data (e.g., vegetation, rainfall) to predict malaria cases in some areas (27, 28). Lee et al., in another research, applied six ML models (including SVM and CatBoost) in predicting malaria from patient data and had a more than 90% accuracy rate (8).

ML has also helped in analysing the impact of environmental conditions. A model by Kim et al. proved to have superior short-term prediction by correlating pollution events with seasonal temperature, 23–24 °C (29). Such predictive models are a goldmine for prevention measures and resource allocation in risk-prone environments (30, 31).

### 4.3 Commonly used ML algorithms

Public health algorithms include Support Vector Machines (SVM), Naive Bayes (NB), Random Forests (RF), and k-Nearest Neighbor Classifier (k-NNC) (21, 23). Data type, complexity of classification, and interpretability needs decide the algorithm. RF is typically used for ensemble prediction, while SVM is optimal for binary classification in high-dimensional data (30, 32).

### 5 AI/ML in infectious disease surveillance with social media and search data

**Figure 3:**
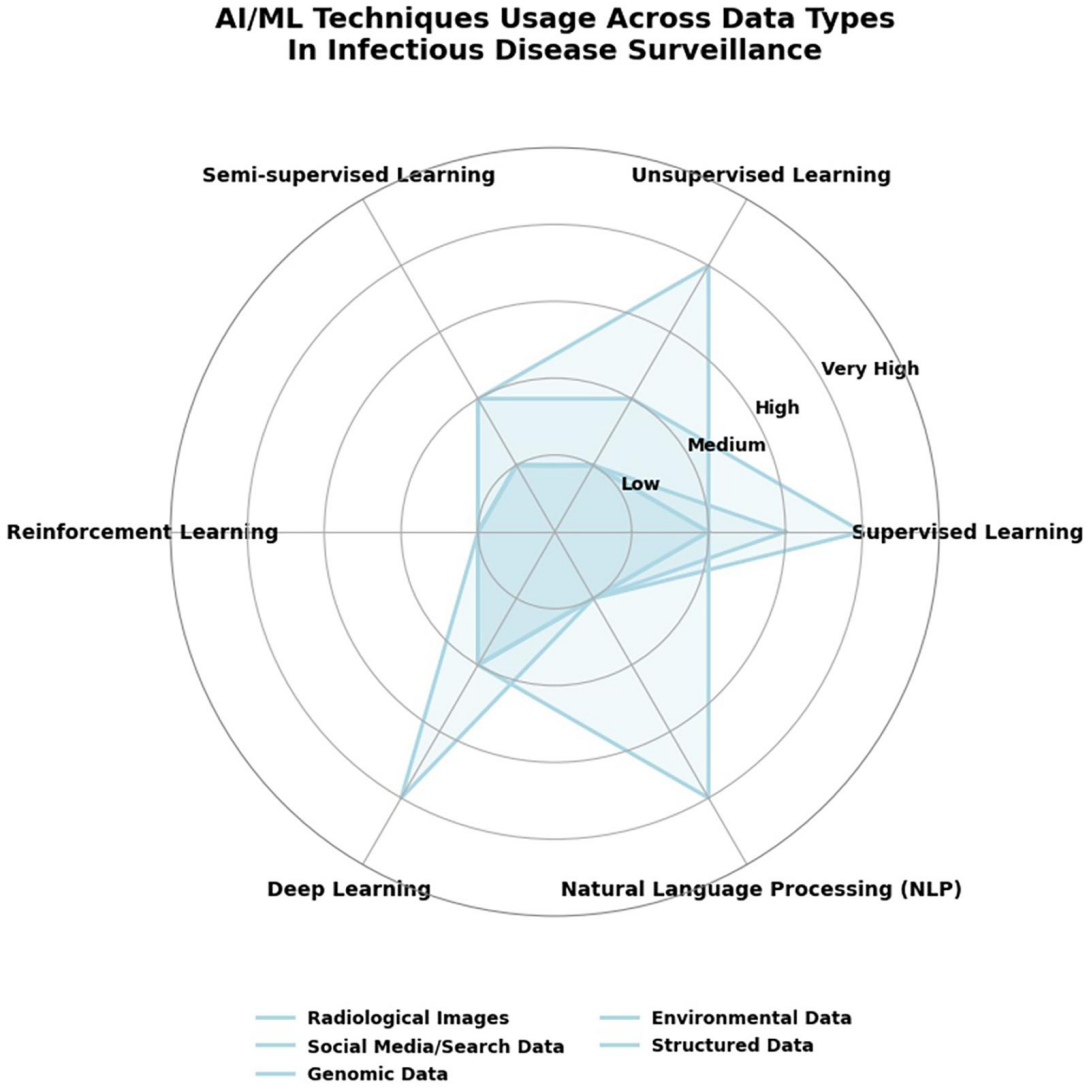
AI/ML techniques usage across data types in infectious disease surveillance.

Artificial Intelligence (AI) and Machine Learning (ML) techniques have become more prevalent in disease surveillance and forecasting of infectious diseases based on large-scale, real-time data streams. A prime example is Santillana et al.'s (3) creation of an ensemble machine learning model that integrates Google search data, X microblog data (previously Twitter), real-time hospitalization data, and participatory surveillance systems for Influenza-like Illness (ILI) activity prediction in the US (32, 33). Their model was predictively accurate, better than models based on any single data stream, and provided forecasts four weeks in advance of official CDC reports. Adding to this, Signorini et al. (33) demonstrated the utilization of real-time Twitter data not just to track disease occurrence during the H1N1 pandemic but to quantify public concern (33, 34). These studies show how supervised ML and NLP can be applied to examine vast levels of unstructured social media and query search data to provide timely, population-level disease surveillance intelligence (35). This is in keeping with more general AI/ML techniques, e.g., NLP and ensemble learning, that allow for early detection of outbreaks, public sentiment estimation, and quick response in public health systems, as shown in Figure 3 (18, 21, 32).

## 6 Challenges

Application of big data analytics in the healthcare sector is transformative in potential, particularly in times of health crises like the COVID-19 pandemic. However, as shown in Table 1, several challenges become impediments to its proper application and impact.

**Table 1:** Challenges in implementing big data analytics in healthcare.

Challesnge	Description
1. Patient Data Confidentiality and Security	Patient data confidentiality and security are key concerns to healthcare officials as well as the public in general. Medical information should be made available only under stringent circumstances and to trained experts or researchers. Having solid data access frameworks with legal and ethical requirements is paramount to protect against misuse or unauthorized release. This line of thinking is especially critical in crisis scenarios like COVID-19 when a balance between expediency of data access must be balanced against the risk of violating patient confidentiality (46).
2. Data Sharing and Interoperability	The power of big data is information volume and variety. However, barriers to data sharing such as institutional silos, incompatible systems, and mistrust might limit its value. During the initial phase of the COVID-19 pandemic in Wuhan, there was a global concern regarding the availability and trustworthiness of information in shared data. Blockchain technologies have been proposed as secure solutions for large-scale, anonymized data sharing that could give transparency, traceability, and integrity of data while respecting patient confidentiality (46).
3. Misinformation and Data Validity	As much as digital media enable instant information exchange, they are also hotspots for medical misinformation and gossip. In pandemics, dissemination of false news thwarts public health initiatives, misleads the general populace, and leads to harmful practices. Additionally, defective or inaccurate data in studies may manipulate results. Big data and artificial intelligence technologies can be employed to detect misinformation, verify online content, and filter out unreliable sources so that policymakers and the public are working with accurate information (46).
4. Patient Cooperation and Data Provision	Healthcare based on data also depends on patient cooperation. Exposure of personal health data, such as wearables' real-time data and medical history, can enhance predictive models and early warning systems. However, the majority of individuals are reluctant to share such information due to privacy issues. For instance, a poll conducted in January 2020 indicated that just 37% of 4,600 healthy study participants would be willing to share their health data with research institutions. It is of great importance to create public awareness about the utility of de-identified (blind) sharing of information and clearly inform individuals about who can collect and use such information in order to gain people's trust and foster involvement.
5. Manager and Technical Considerations	In order for a healthcare big data analytics platform to be successful, it must offer essential abilities of real-time processing, scalability, interoperability, and robust security controls. Most platforms available today are open-source, and they vary in terms of usability, dependability, and flexibility. Platforms must be simple to use, have simple-to-use interfaces, drop-down menus for selecting algorithms, and real-time analytics abilities. Moreover, managerial problems like governance, data ownership, and compliance with data standards must be addressed. Constant acquisition of data and rigorous data cleaning must be done as well, since healthcare data are likely to be fragmented, non-standardized, and stored in legacy systems (1, 2, 47).

## 7 Policy recommendations

There are different indicators of infectious disease preparedness that decision-makers need to investigate when evaluating a health system's readiness (36–38). Indicators of such concern could be the impact of emergencies on equity; core public health, and government capacities for emergency preparedness and response; vulnerabilities in the population and healthcare system during pandemics; community readiness, and benchmarks for strengthening health systems during outbreaks. On the other hand, there are indicators related to public health and health system readiness or capacity: adequate public health budget; capacity to deliver vaccines; the proportion of the population getting vaccinated; licensed healthcare professionals, especially nurses, and their ability to practice in other regions or states; oversight of research on dangerous pathogens; and enhanced training for the safe transportation of biohazards. Adopting and institutionalizing ethical digital development principles for AI in global health interventions is, without a doubt, an area to touch upon and requires proper policy recommendations and actions, as shown in Table 2 (39–42).

**Table 2:** Policy options and considerations for public health emergency preparedness (8, 12, 20, 35, 37–39, 41, 43, 48).

Policy options	Monitoring of preparedness indicators	Strengthening health system capacity	Adpoting equity-focused preparedness	Enhancing governance and legislation	Implement digital health & AI principles
Description	Create measures to track health system readiness, emergency impact on equity, community preparedness, and health system vulnerabilities.	Invest in public health budgets, vaccine delivery infrastructure, flexibility in the workforce, and safe pathogen research management.	Prioritizes social determinants such as access to technology, housing, clean water, and specially tailored plans for the disadvantaged.	Establish emergency roles, responsibilities, and enables timely decision-making; incorporate environmental health considerations.	Develop user-interaction-based AI and digital interventions based on scalability, sustainability, data-driven action, open collaboration, and privacy/security protection.
Advantages	Provides a general overview of system vulnerabilities and strengths	Enhances response speed and coverage.	Suppresses health inequities and improves outcomes in at-risk populations.	Strengthens system accountability and coordination.	Fosters effective, ethical, scalable digital health interventions.
Disadvantages	Requires robust data collection and continuous updating	Can suffer from funding and workforce training challenges.	Requires cross-sectoral effort and targeted outreach.	Legal change may be politically difficult and time-consuming.	Requires technical capability and ongoing funding.
Cost & feasibility	Moderate cost; requires investment in information systems and analysis; feasible with existing health info systems.	Costly; feasible long-term with political will and investment in resources.	Moderate to high cost depending on upgrade of social infrastructure; achievable with multi-sector collaboration.	Variable cost; requires legislative processes; medium feasibility depending on political will.	Moderate development and maintenance investment; viability dependent on infrastructure and collaboration.
Equity Considerations	Requires indicators relevant to vulnerable and marginalized groups.	Needs to prioritize underserved areas and offer access equity.	Points efforts toward equity gaps; must be careful to avoid unintended exclusion.	Must ensure that laws promote fair access to emergency services and protections.	Requires inclusive design not to reinforce digital divides; requires protection of sensitive information for at-risk populations.
Stakeholders	Public health practitioners, policymakers, data analysts, community leaders.	Health ministries, hospitals, training institutions, regulatory agencies.	Public health agencies, social services, community organizations, policymakers	Legislators, public health officials, legal practitioners, environmental agencies	Developers of technology, public health communities, funders, community leaders, data privacy administrators.

## 8 Discussion

To focus on infectious disease preparedness, equity-related preparedness indicators are crucial. They are envisaged in the proportion of the racialized or first-generation immigrant population in a defined region; benchmarks for public health agency plans to address the needs of racialized or marginalized populations; the proportion of the population with access to internet and technology; the ratio of residential and nursing homes per 10,000 population aged over 70 years old, proportion of population with access to clean water, and the proportion of households with inadequate living conditions.

Climate and environmental health, as well as public health legislation, are areas that have gained renewed attention in relation to preparedness and in light of the COVID-19 pandemic. Governance and leadership are fundamental elements in the Resilience Framework for Public Health Emergency Preparedness (43–46).

To ensure that artificial intelligence (AI) and digital technologies are introduced in a way that is ethical, equitable, and sustainable, health systems and global institutions need to integrate a core set of digital development principles into their national and regional digital health policies:

1. Human-centered design policy that should demand engagement of end users and communities in designing digital interventions;
2. Contextual adaptation in AI solutions should consider country- and community-level structures, capacities, and needs;
3. Scalability in terms of collaboration and funding models should be included in policy to enable scaling up successful pilots;
4. Sustainability in long-term maintenance and stakeholder engagement should be planned from the onset;
5. Data-driven action that necessitates proper infrastructure to provide high-quality data that is available, accessible, and usable for decision-makers;
6. Openness and collaboration, open-source frameworks, and knowledge sharing between sectors should be promoted by funders and governments;
7. Reusability and improvement policies should support the reuse of current tools rather than redundant new development;
8. Privacy and security, along with strong data governance frameworks, should be established that outline how data is gathered, stored, and shared;
9. Multi-sectoral coordination policies must promote joint planning and implementation across health, tech, and civil society actors.

## 9 Future directions

### 9.1 Human-centric AI, privacy, and governance

The aim we are reaching for with artificial intelligence technology and algorithm is a human-centered solution. By their nature, privacy and governance are related and harder to manage; nevertheless, serious efforts are being made both by governments and by manufacturing organizations, even with a mysterious blind spot. A cooperative effort with interested stakeholders, informed by a combination of trial-and-error processes and studies, is needed to identify a potential candidate for investigation and maturation.

### 9.2 Inclusion, equity, and societal impacts

The adoption of inclusion and equity studies and their related applications is not yet fully established in some countries, and this can lead to discrimination and lower applicability, as users can be excluded by AI programs or wrongly classified according to various characteristics and profiles. Value and social impact studies are also imperative for the aggregation of overall advantages and the reduction of opportunity cost related to other choices. By conducting these studies, AI companies and public agencies can control market entry and ensure value creation throughout the whole lifespan of AI technologies.

### 9.3 AI design with a human-oriented approach

Studies and analysis related to human-in-the-loop, human-on-the-loop, and human-in-command approaches are required to promote the adoption of such methods in early stages of AIalgorithm and machine learning code building; therefore, allowing for utilization to remain responsible and accountable.

### 9.4 Healthcare and challenges

Patient acceptability, participation, and compliance have been termed the “last mile” challenge in healthcare as the ultimate hurdle to improving health outcomes. They are now also being solved with big data and machine learning. And user-friendliness, along with scalability, are also key requirements for mass deployability. Hospitals and providers typically fall back on their clinical experience to create treatment protocols that work to reduce adverse outcomes for chronic and acute patients. Non-compliance by patients is still a major challenge.

### 9.5 Explainability and transparency in AI

Among the most critical problems currently is that of clarity: Deep Learning models used for image analysis are fundamentally opaque and immune to simple explanation or interpretation. When a patient is told that an image aided in a diagnosis of cancer, they will undoubtedly ask for an explanation about the rationale behind it. Furthermore, even experienced medical professionals will find difficulty providing articulate explanations.

### 9.6 Adoption challenges in clinical practice

The bigger challenge facing AI in health care is not whether the technology will become powerful enough, but how to achieve adoption in daily clinical and public health practice. AI programs will have to be licensed by authorities, instructed to physicians, standardized appropriately, incorporated with electronic health record programs, and kept up to date on a continuous basis; they are all necessities for broad adoption. They will also have to be paid for by public and private payers.

### 9.7 Data management and disease-specific AI challenges

Greater work is needed in data administration, that is, standardizing the numerous data sources prevalent in health organizations and health infrastructure. Unique issues are presented by each novel and re-emerging infectious disease.

### 9.8 Regulation and deployment

Whereas many companies that focus on developing artificial intelligence are currently hastening the transition from design to deployment, this inevitably leads to a series of fallibilities, mostly based on societal or human grounds. Time must be taken to devise the right regulations to govern such technologies. Finally, work must be geared towards enhancing the preparedness and interoperability of nations across the globe to effectively integrate artificial intelligence and machine learning technologies into their healthcare schemes.

## 10 Conclusion

This review is aimed at policymakers, decision-makers, and organizations seeking guidance on trends at the intersection of AI/ML and public health surveillance. The primary goal of Public health informatics is to safeguard global public health. What's to be stressed is the urgency of keeping pace with the technological breakthroughs in today's health tech era. The governing and leading role of global public health within the healthcare hierarchy is undeniable and must be backed with all available advancements, while ensuring proper utilization and ethical practices. COVID and other pandemics have shown us how to adapt new tools to foster collaboration, or in other words, how to humanize our efforts and tackle hurdles and roadblocks as a united front. The essentiality of integrating health and technology is dire and daunting. Public health informatics tools are vital in controlling infectious diseases. In conclusion, one can sum up with the conviction that strides have been made in this rapidly growing multidisciplinary domain of AI technology, which remains in its early stages; nonetheless, there is still much more to explore and develop, with curiosity and caution. At last, the alarming need for AI-enabled and AI-enhanced public health surveillance and emergency preparedness systems in our health systems is clear.
